# Physiological Implications of Hydrogen Sulfide in Plants: Pleasant Exploration behind Its Unpleasant Odour

**DOI:** 10.1155/2015/397502

**Published:** 2015-05-11

**Authors:** Zhuping Jin, Yanxi Pei

**Affiliations:** ^1^School of Life Science, Shanxi University, Taiyuan 030006, China; ^2^School of Chemical Engineering and Environment, North University of China, Taiyuan 030051, China

## Abstract

Recently, overwhelming evidence has proven that hydrogen sulfide (H_2_S), which was identified as a gasotransmitter in animals, plays important roles in diverse physiological processes in plants as well. With the discovery and systematic classification of the enzymes producing H_2_S *in vivo*, a better understanding of the mechanisms by which H_2_S influences plant responses to various stimuli was reached. There are many functions of H_2_S, including the modulation of defense responses and plant growth and development, as well as the regulation of senescence and maturation. Additionally, mounting evidence indicates that H_2_S signaling interacts with plant hormones, hydrogen peroxide, nitric oxide, carbon monoxide, and other molecules in signaling pathways.

## 1. Introduction

Hydrogen sulfide (H_2_S) is a colorless, flammable gas with the characteristic odor of rotten eggs. It was widely considered to be just a toxic gas for nearly 300 years mostly due to its unpleasant smell. The breakthrough in the effort to link endogenous H_2_S levels and functional changes came when the possible role of H_2_S as an endogenous neuromodulator in the brain was reported [1]. The focus on enzymes generating H_2_S was another breakthrough in 2001 [2]. The initial work concluded that H_2_S was a physiological vasodilator and regulator of blood pressure, which stimulated research on H_2_S physiology [3]. In plants, H_2_S has been revealed as a crucial player in the regulation of normal plant physiological processes, including seed germination, root morphogenesis, photosynthesis, and flower senescence [4–8]. It was also shown to be an important messenger in plant defense signaling against various abiotic stresses at physiological concentrations [9–13]. In this review, we discuss recent progress that increases our understanding of H_2_S synthesis and signaling functions in plants.

## 2. H_**2**_S Synthesis

**Figure 1:**
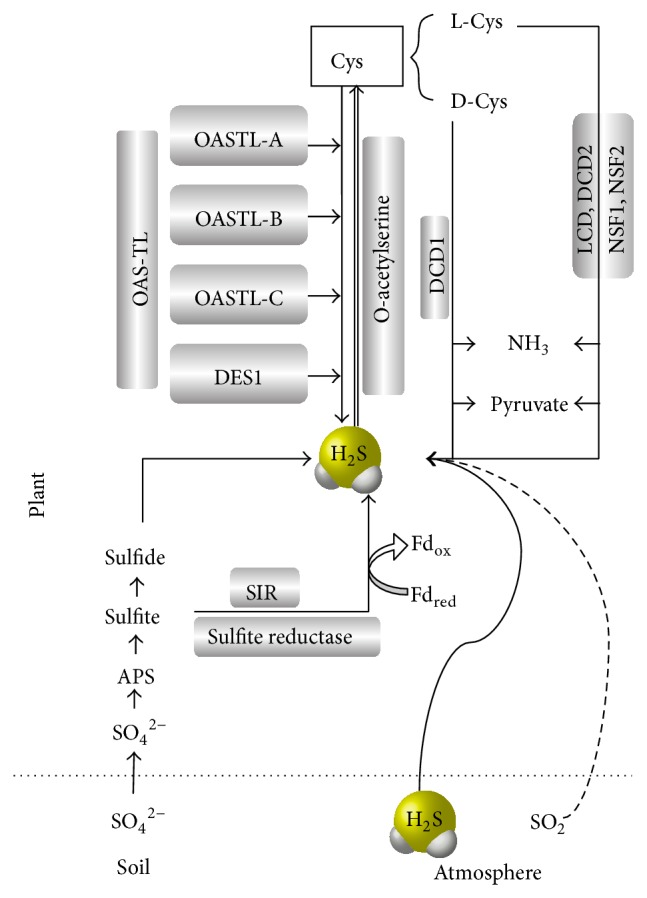
An overview of H_2_S generation in higher plants (adapted from Papenbrock et al., 2007). APS: adenosine 5′-phosphosulfate; Fd_red_, Fd_ox_: reduced and oxidized ferredoxin; SIR: sulfite reductase.

In mammalian cells, H_2_S is physiologically generated by pyridoxal-5′-phosphate-dependent enzymes, including cystathionine beta-synthase, cystathionine gamma-lyase, and 3-mercaptopyruvate sulfurtransferase (3-MST), during cysteine (Cys) metabolism [3, 14]. H_2_S is generated in plants via both enzymatic and nonenzymatic pathways, although the latter only accounts for a small portion of H_2_S production.  Figure 1, with the enzymes highlighted, demonstrates the production of H_2_S in* Arabidopsis thaliana*.

Several candidate Cys-degrading enzymes have been reported to exist in different plant species (shown in Table 1). In the model plant* A. thaliana*, the enzymes that produce H_2_S can be roughly divided into two categories. One class of these enzymes is Cys desulfhydrases (CDes), which degrade Cys into H_2_S, ammonia, and pyruvate in a stoichiometric ratio of 1 : 1 : 1 and require pyridoxal 5′-phosphate as a cofactor [15]. L-Cys desulfhydrase is one of the enzymes that decompose L-Cys and was first discovered in the sulfur metabolism of tobacco cultured cells [16]. D-Cys desulfhydrase 1 specifically uses D-Cys as its substrate, and D-Cys desulfhydrase 2 degrades L/D-Cys simultaneously [17, 18]. The production of H_2_S by CDes has been confirmed in various areas of biology [9, 11, 14, 15, 19, 20]. CDes are Cys desulfhydrases with singular functions in desulfuration. Their mRNA levels were significantly higher in the stems and cauline leaves than in the roots, rosette leaves, and flowers of* A. thaliana* [9].

Another class of the enzymes is O-acetyl-L-serine (thiol) lyase (OAS-TL), which is responsible for the incorporation of inorganic S into Cys, and free H_2_S appears to be released only in a minor reaction [21]. During an incubation period, the enzyme formed about 25 times more Cys than H_2_S, in a molar ratio, per mg protein [22]. Nine OAS-TL genes have been identified in* A. thaliana*, which are located in the cytosol, mitochondria, or plastid [23]. Recently, DES1 was reported as a frequent novel L-Cys desulfhydrase, which, based on sequence feature alignments, belongs to the OAS-TL family [24–28]. The* Km* value for L-Cys in the DES1 reaction is 13-fold lower than that for OAS in the OAS-TL reaction, indicating a much higher affinity of DES1 for L-Cys as a substrate [2]. The biochemical characterization of the T-DNA insertion mutant* des1* reveals that the total intracellular Cys concentration increased by approximately 25% [28]. However, as a member of the OAS-TL family, its function in synthesizing H_2_S has not been clearly studied.* In vitro*, the reaction of OAS-TL is a net H_2_S-consuming reaction [22]. Thus, the statement that DES1 is the only enzyme involving in the degradation of Cys is open to question [24, 28, 29].

In addition, Nifs/NFS, with L-Cys desulfhydrase-like activity, is also potentially involved in H_2_S production [31, 32]. Two genes, At5g26600 and At1g01010, in* A. thaliana* have been identified that encode proteins with CDes structural features [15], and 3-MST is also related to H_2_S production in plants [33].

## 3. Physiological Functions of H_**2**_S in Plants

H_2_S has been reported to play important roles in diverse physiological processes in plants. Research on the endogenous H_2_S of higher plants can be traced back to 1978, when H_2_S was observed to be released from leaves of cucumber, corn, and soybean [34]. Leaves of older plants contain higher H_2_S concentrations than younger plants [35]. A recent study showed that the mRNA levels of* CDes* were gradually elevated in a developmental stage-dependent manner [9]. The importance of H_2_S in the regulation of plant growth, development, and senescence has emerged.

The improvement in seed germination rates due to exogenous H_2_S treatments was confirmed. H_2_S or HS^−^, rather than other sulfur-containing components derived from the exogenous H_2_S donor, NaHS, contributed to the promotion of seed germination [4]. NaHS preferentially affects the activity of endosperm *β*-amylase and maintains lower levels of malondialdehyde and hydrogen peroxide (H_2_O_2_) in germinating seeds [7]. In addition, the application of NaHS to seedling cuttings of sweet potato promoted the number and length of adventitious roots [5]. At the same time, H_2_S modulates the expression of genes involved in photosynthesis and thiol redox modification to regulate its photosynthesis [36]. It is hypothesized that an increase in the stomatal density also contributes to this process [37]. The osmotic-induced decrease in the chlorophyll concentration could be alleviated by spraying the NaHS solution [6]. H_2_S was also found to delay flower opening and senescence in cut flowers and branches [8]. These effects occur in a dose-dependent manner. In the cytosol, H_2_S negatively regulates autophagy and modulates the transcriptional profile of* A. thaliana* using* des1* [38]. H_2_S strongly affects plant metabolism at most stages of life and causes statistically significant increases in biomass, including higher fruit yields [39].

H_2_S also plays pivotal roles in plant responses or adaptation under biotic and abiotic stress conditions. Early studies concerning H_2_S emissions in plants were associated with plant responses to pathogens as part of sulfur-induced resistance [40]. In 2008, H_2_S was found to be an important cellular signal for the first time, highlighting the protective effect of H_2_S against copper stress [4]. Thereafter, a stream of publications on various positive effects of H_2_S and H_2_S signaling in plants emerged. Soon, H_2_S was shown to alleviate the effects of aluminum, cadmium, chromium and boron toxicity, drought and osmotic stress, heat stress, hypoxia, and other stresses [9, 11–13, 20, 41–43]. Most of these reports discussed, as analogies with animal systems, how H_2_S signaling is important for plant protection against stress.

Stomatal movement is very important in plant responses to environmental stimuli, and a key target of H_2_S signaling in plants is the specialized guard cell. Recent studies have reported that H_2_S is responsible for drought stress relief by inducing stomatal closure in* A. thaliana* [9, 20]. These observations are consistent with a previous report in both* Vicia faba* and* Impatiens walleriana* [30]. Similarly, H_2_S was confirmed to be a novel downstream indicator of nitric oxide (NO) during ethylene-induced stomatal closure [44]. However, the effect of H_2_S on stomatal movement has been a controversial topic. Another research group reported that exogenous H_2_S induced stomatal opening by reducing the accumulation of NO in guard cells of* A. thaliana* and a crop plant,* Capsicum annuum* [45, 46]. The reasons for these different observations are not clear and require further study. The difference may simply be due to the different experimental materials and methods. The purpose of stomatal closure is to reduce the moisture loss under drought stress, and the induction of stomatal opening is to enhance photosynthesis and reduce the photorespiration.

## 4. Cross-talk of H_**2**_S with Other Signals

**Figure 2:**
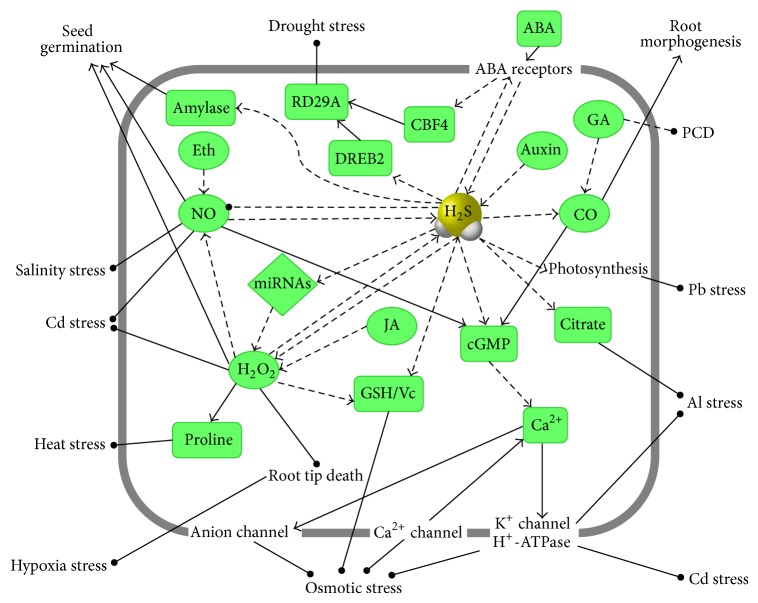
Generalized model of H_2_S signaling in response to abiotic stress in plants. Solid line arrows depict stimulatory effect; dashed cents arrows represent the putative interaction; gray bold rectangle indicates cell membrane. ABA: abscisic acid; CBF: C-repeat binding factor; CO: carbon monoxide; DREB: dehydration responsive element; Eth: ethylene; GA: gibberellic acid; cGMP: cyclic guanosine monophosphate; GSH: glutathione; H_2_O_2_: hydrogen peroxide; H_2_S: hydrogen sulfide; JA: jasmonic acid; NO: nitric oxide; PCD: programmed cell death; RD: responsive to desiccation.

Plants perceive and respond to H_2_S, but studies on the mechanisms of H_2_S functioning in plant responses to stress are very limited. An overview of our current understanding of plant H_2_S signaling is shown in Figure 2. H_2_S is particularly active and may interact with and modify numerous other signals. Thus, there may be multiple routes of H_2_S perception and signaling to be unraveled.

Several lines of evidence point to an interrelationship between H_2_S and plant hormones in plant defenses. Abscisic acid (ABA) is produced in large amounts in plants under various abiotic stresses. Under drought stress, the expression of* CDes* was significantly upregulated, and the production rate of H_2_S from these plants also increased [9]. Subsequently, the relationship between H_2_S and ABA was reported based on a deficiency of H_2_S in the* lcd* mutant that had a weakened ABA induction of stomatal closure, which indicated that the induction of stomatal closure by ABA was partially dependent on H_2_S. As H_2_S was also involved in the expression regulation of ion-channel genes, H_2_S may be a critical component of ABA-induced stomatal closure via ion channels. At the same time, H_2_S influenced the expression of ABA receptors, and the influence of H_2_S may have begun upstream of the ABA signaling pathway. Therefore, the above results showed that H_2_S interacted with ABA in the stomatal regulation responsible for drought stress in* A. thaliana* [20]. Indole acetic acid (IAA) showed a rapid increase in different plants treated by exogenous H_2_S [5], and ethylene (Eth) could induce H_2_S generation [44]. In addition, gibberellic acid (GA) and jasmonic acid (JA) were also involved in the H_2_S signal transduction process. H_2_S can alleviate the GA-induced programmed cell death in wheat aleurone cells [47], and H_2_S may function downstream of H_2_O_2_ in JA-induced stomatal closure in* V. faba* [48].

H_2_O_2_ is another signaling molecule in plants, especially in guard cells. Abiotic stress induces synthesis of both H_2_S and H_2_O_2_; yet it is unclear how these two molecules work in concert in the physiological process. H_2_S may represent a novel downstream component of the H_2_O_2_ signaling cascade during JA-induced stomatal movement in* V. faba* [48]. Pretreatment of H_2_O_2_ could improve the germination percentage of* Jatropha curcas* seeds, and this improvement was mediated by H_2_S [49]. These results suggest that H_2_O_2_ is upstream of H_2_S. However, there is plenty of evidence to the contrary. H_2_S inhibited the cadmium influx through the plasma membrane calcium channels, which were activated by H_2_O_2_ [50]. H_2_S can participate in enhancing plant resistance to abiotic stress via the improvement of antioxidant systems, such as heavy metal stress, osmotic stress, heat stress, and hypoxia stress [4–7, 10, 42, 43, 49].

Recent evidence suggests that H_2_S also plays a role in the NO and carbon monoxide (CO) signaling pathway. In bermudagrass, sodium nitroprusside (SNP, a NO donor) and NaHS combined treatments showed that NO signaling could be blocked by H_2_S inhibitors and scavengers, indicating that NO-activated H_2_S was essential for the cadmium stress response [51]. Additional evidence showed that both NaHS and GYY4137 reduced the NO accumulation to a large extent in* A. thaliana* epidermal cells [45]. In sweet potato seedlings, a rapid increase in endogenous H_2_S and NO was sequentially observed in shoot tips treated with NaHS. A similar phenomenon in H_2_S donor-dependent root organogenesis was observed in both excised willow shoots and soybean seedlings. These results indicated that the process of H_2_S-induced adventitious root formation was likely mediated by IAA and NO and that H_2_S acts upstream in IAA and NO signaling transduction pathways [5]. Similarly, heme oxygenase 1 functions as a downstream component in H_2_S-induced adventitious root formation by the modulation of expression of related genes, which suggested that CO was involved in H2S-induced cucumber adventitious root formation [52].

Additionally, growing evidence suggests that H_2_S signaling interacts with calcium (Ca) signaling pathways. Ca^2+^ confers structure and rigidity to the cell wall and regulates plant processes through calmodulin. Li et al. (2013) showed that NaHS pretreatment could improve the entry of extracellular Ca^2+^ into tobacco suspension cultured cells mediated by intracellular calmodulin to increase the heat tolerance [41]. At the level of transcription, the expression of Ca^2+^ channel coding genes decreased, whereas Ca^2+^-ATPase and Ca^2+^-H^+^ cation antiporters were elevated in the* lcd* mutant. This was in accordance with stronger Ca^2+^ fluorescence in the wild type than in the* lcd* mutant [20]. These results suggest that Ca signaling plays an important role in the mechanism of H_2_S.

Numerous studies showed that, during the enhancement of plant resistance, many substances changed simultaneously. H_2_S plays an ameliorative role in protecting plants by increasing the proline content against aluminum toxicity and heat stress [10, 12, 41]. Aluminum-induced citrate secretion was also significantly enhanced by NaHS pretreatment [10]. During the NaHS preincubation period the grain *β*-amylase activity increased, improving seed germination [7].

## 5. Conclusions and Perspectives

The mechanisms by which H_2_S is generated still remain unresolved, and elucidating how it is made by different plant cells under different conditions is clearly a research priority. H_2_S is a key factor in the tolerance of cells to the oxidative stress induced by a range of abiotic conditions, including heavy metal toxicity, drought and osmotic stress, hot stress, hypoxia and other stresses. This probably involves the activation of antioxidant defenses, the induction of stomatal closure, and the enhanced expression of genes encoding resistance-associated enzymes. In these processes, plant hormones, H_2_O_2_, NO, CO, and Ca signaling participate in H_2_S signal transduction, resulting in a complex signaling network.

There are numerous unanswered questions and important areas for further research, concentrated in the following areas. (1) Owing to the promiscuous chemical properties of H_2_S, it is problematic to achieve adequate specificity and selectivity for its measurement. At present, the physiological H_2_S level was measured by various techniques such as the methylene blue method, monobromobimane, gas chromatography, ion selective electrodes, and fluorescent probes [53]. The diverse detection methods resulted in magnitude differences in measured biological sulfide levels, which will certainly attract increasing attention. (2) The mechanism of H_2_S functions performed at the protein level. Until now, a great number of studies focused on protein S-sulfhydration, which is impossible to determine directly by chemical analyses. But in mammals, there have been many results indicating that this process might occur by the transition of intermediate links, such as positional changes and interactions with associated proteins. Moreover, if H_2_S can thiolate proteins, it may have the same effect on DNA. (3) Even though H_2_S is a short-lived molecule, it is an extremely active one. The mechanisms by which either H_2_S or other molecules participating in H_2_S signaling function are also important. Thus, elucidation of the H_2_S complex signaling network is clearly a research priority.

## Figures and Tables

**Table 1:** Enzymes and coding genes related to H_2_S generation in *Arabidopsis*.

Substrate	Enzyme	Cellular localization	Locus	Reference
L-Cys	AtLCD	Cytoplasm	At3g62130	[16]
	AtNFS1/Nifs1	Mitochondria	At5g65720	[27]
	AtNFS2/Nifs2	Plastid	At1g08490	[28]
	DES1	Cytoplasm	At5g28030	[24]
	OASTL-A1	Cytoplasm	At4g14880	[25]
	OASTL-A2	Cytoplasm	At3g22460	[30]
	OASTL-B	Plastid	At2g43750	[26]
	OASTL-C	Mitochondria	At3g59760	[27]

D-Cys	AtDCD1	Mitochondria	At1g48420	[17]

L/D-Cys	AtDCD2	Mitochondria	At3g26115	[18]

Unknown	PLP-dependent transferase superfamily	Chloroplast	At5g26600	[15]

Unknown	NAC domain containing protein 1	Unknown	At1g01010	[15]

At:* Arabidopsis thaliana*, Cys: cysteine, DCD: D-Cys desulfhydrase, DES: desulfhydrase, LCD: L-Cys desulfhydrase, NAC: N-acetyl-L-cysteine, NFS: nitrogenase Fe-S cluster, OASTL: O-acetyl-L-serine(thiol)lyase, and PLP: pyridoxal 5′-phosphate.
